# Comparing general and regional anesthesia in patients undergoing primary total hip arthroplasty: analysis of national health insurance data in Korea

**DOI:** 10.3389/fmed.2025.1557053

**Published:** 2025-03-17

**Authors:** Seungyoung Lee, Eunjin Ahn, Min Kyoung Kim, Fletcher A. White, Euiheon Chung, YongHun Chung

**Affiliations:** ^1^Department of Anesthesiology and Pain Medicine, Chung-Ang University Hospital, Seoul, Republic of Korea; ^2^Department of Anesthesiology and Pain Medicine, Chung-Ang University Gwangmyeong Hospital, Gwangmyeonsi, Republic of Korea; ^3^Department of Anesthesiology and Pain Medicine, College of Medicine, Chung-Ang University, Seoul, Republic of Korea; ^4^School of Medicine, Stark Neuroscience Research Institute, Indiana University Bloomington, Indianapolis, IN, United States; ^5^Department of Anesthesia, Indiana University School of Medicine, Indianapolis, IN, United States; ^6^Department of Family Medicine, Veterans Health Service Medical Center, Seoul, Republic of Korea

**Keywords:** anesthesia, hip arthroplasty, mortality, surgery, complications

## Abstract

**Objectives:**

To compare the effects of general and regional anesthesia on clinical outcomes following primary total hip arthroplasty (THA).

**Methods:**

This retrospective study using data from the Korean National Health Insurance Research Database included 1,522 patients who underwent THA under general anesthesia (*n* = 640) or regional anesthesia (*n* = 882) between 2002 and 2015. We compared the mortality and complication rates within 30 days after surgery.

**Results:**

Prosthesis failure (1.56% vs. 0.45%, *p* = 0.025), admission to the intensive care unit (9.53 vs. 5.44%, *p =* 0.0023), and total cost (₩7,332,515 vs. ₩6,833,295, *p* < 0.0001) were higher in the general anesthesia group than in the regional anesthesia group. No significant differences were observed in mortality (0.94% vs. 0.57%, *p* = 0.54), transfusion rate (81.1% vs. 80.9%*, p* = 0.94), length of hospital stay (45 vs. 45 days, *p* = 0.23), or other complications between the groups. Similar results were observed in propensity-score matched analysis (*n* = 640 patients per group).

**Conclusion:**

Our study showed that both anesthesia types resulted in comparable mortality and complication rates in patients who underwent THA, but the costs differed.

## Introduction

Total hip arthroplasty (THA) is a surgical intervention commonly indicated for end-stage hip osteoarthritis (1). Owing to population ageing, the number of THA performed is continuously increasing (2, 3). Compared to younger patients, the risk of THA-associated postoperative complications is higher in older patients (2), with a high mortality rate of 5.8% (4).

Numerous studies are currently investigating modifications in THA surgical and anesthesia strategies to reduce postoperative mortality (4–7). However, the effects of anesthesia on clinical outcomes remain poorly understood, as conflicting results have been reported (4, 5). A study involving a large sample size showed that regional anesthesia (RA) was associated with a lower mortality rate and fewer complications, including delirium, whereas another study found that the mortality rates associated with general anesthesia (GA) and RA were comparable (4, 5).

Therefore, in this study, we aimed to compare the effects of GA and RA on clinical outcomes in patients who underwent THA based on the associated mortality and complication rates, using sample data provided by the National Health Insurance Service in Korea. Additionally, we aimed to analyze the changes in anesthesia methods and institutions performing THA surgeries in Korea.

## Methods

This retrospective study was reviewed and approved by the Institutional Review Board (IRB No. 2210-032-047), and the need for informed consent was waived because de-identified administrative data was used in this study. We analyzed data of patients who underwent THA between 2002 and 2015 obtained from the National Health Insurance Service-National Sample Cohort (NHIS-NSC). Patients were selected from the NHIS-NSC database using surgical codes for THA (N0711 and N2070) and anesthesia codes of GA (L1211, L1212, L0101, and L0103) and RA (L1213, L1214). The NHIS serves as the primary mandatory national healthcare institution, with approximately 97% of Korean citizens enrolled in the service. The NHIS-NSC database contains information from 1,025,340 randomly selected individuals, representing approximately 2.2% of the total Korean population (8).

In Korea, hospitals are categorized into clinics, hospitals, general hospitals, and tertiary hospitals. Clinics primarily focus on outpatient care but are legally permitted to have up to 29 inpatient beds. Hospitals must have a minimum of 30 inpatient beds, whereas general hospitals are institutions with a minimum of 100 inpatient beds that offer specialized physician services in major areas. Tertiary hospitals, are general hospitals approved to provide a wide range of advanced medical care with a minimum of 20 departments, catering to severely ill patients. Therefore, we redefined clinics, hospitals, general hospitals, and tertiary hospitals as primary, secondary, tertiary, and quaternary care hospitals, respectively.

Based on anesthesia type, we categorized the patients into GA and RA groups. GA included volatile and intravenous anesthesia, whereas RA included spinal, epidural, and combined spinal-epidural anesthesia. Demographic characteristics such as sex, age, and hospital type were documented for all patients. Owing to the nature of the data, we could only obtain additional demographic details, including height, weight, and body mass index (BMI), for 1,212 individuals (79.6% of the study population).

The Elixhauser Comorbidity Scores (ECS) (9, 10) were determined to quantify each patient’s comorbidities based on 31 comorbidity conditions encompassing AIDS/HIV infection, alcohol abuse, blood loss anemia, cardiac arrhythmia, chronic pulmonary disease, coagulopathy, complicated diabetes mellitus, complicated hypertension, congestive heart failure, deficiency anemia, depression, drug abuse, fluid and electrolyte disorders, hypothyroidism, liver disease, lymphoma, metastatic cancer, obesity, other neurological disorders, paralysis, peptic ulcer disease, peripheral vascular disorders, psychoses, pulmonary circulation disorders, renal failure, rheumatoid arthritis, solid tumor without metastasis, uncomplicated hypertension, uncomplicated diabetes mellitus, valvular disease, and weight loss.

The primary outcome was 30-day mortality, which was defined as death (in or out of hospital) from any cause within 30 days of index admission. Delirium was defined as the administration of drugs including quetiapine, risperidone, and haloperidol during hospitalization. Transfusion rates, volumes, and types (including red blood cells, fresh frozen plasma, platelets, and whole blood) were examined using transfusion codes. Additional recorded parameters included length of hospital stay (LOS), total cost, and an investigation into postoperative complications such as admission to intensive care unit (ICU), unplanned intubation, ventilator care, cardiac arrest, myocardial infarction, pneumonia, pulmonary edema, acute respiratory distress syndrome, intracranial hemorrhage, stroke, pulmonary thromboembolism, other thromboembolic events, nerve injury, prosthesis failure, wound dehiscence, surgical site infection, sepsis, urinary tract infection, acute renal failure, and hepatic failure.

Normally distributed variables were evaluated using the Kolmogorov–Smirnov or Shapiro–Wilk test. To minimize selection bias, we performed propensity score matching in a 1:1 ratio using the caliper matching method. Propensity scores were calculated for age, sex, and ECS using logistic regression analysis. For pre-matching data, continuous variables were analyzed using the Wilcoxon rank-sum test, whereas categorical variables were compared using the chi-square or Fisher’s exact test. For post-matching data, continuous variables were compared using the paired *t*-test or Wilcoxon signed rank-sum test, whereas categorical variables were compared using the McNemar or exact McNemar test. All statistical analyses were two-sided, and the significance level was set at *p* < 0.05. The R version 3.4.1 (RStudio, Boston, MA, United States) and SAS Enterprise Guide version 6.1 (SAS Institute Inc., Cary, NC, United States) were used for all analyses.

## Results

**Figure 1:**
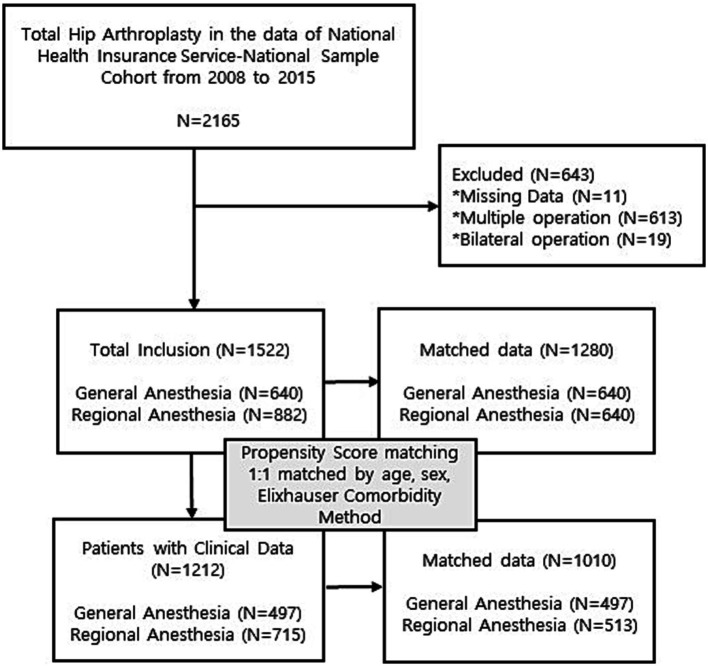
Flow diagram.

We enrolled 2,165 patients who underwent primary THA under GA or RA between January 2002 and December 2015. A total of 643 patients were excluded because of missing data, multiple operations, and staged or simultaneous bilateral operations. After exclusion, 1,522 patients who underwent unilateral primary THA were included in this study. Among 1,522 patients, only 1,212 had available clinical examination data such as height, weight and body mass index (Figure 1).

**Figure 2:**
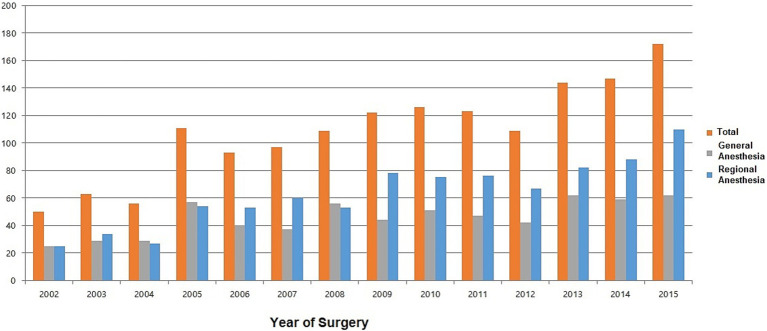
Trends in total hip arthroplasty according to the type of anesthesia.

From 2002 to 2015, THA frequency steadily increased (Figure 2). In addition, analysis of changes in anesthesia methods over the years revealed a steady increase in the use of RA compared with the use of GA from 2002 to 2015 (Figure 2).

**Figure 3:**
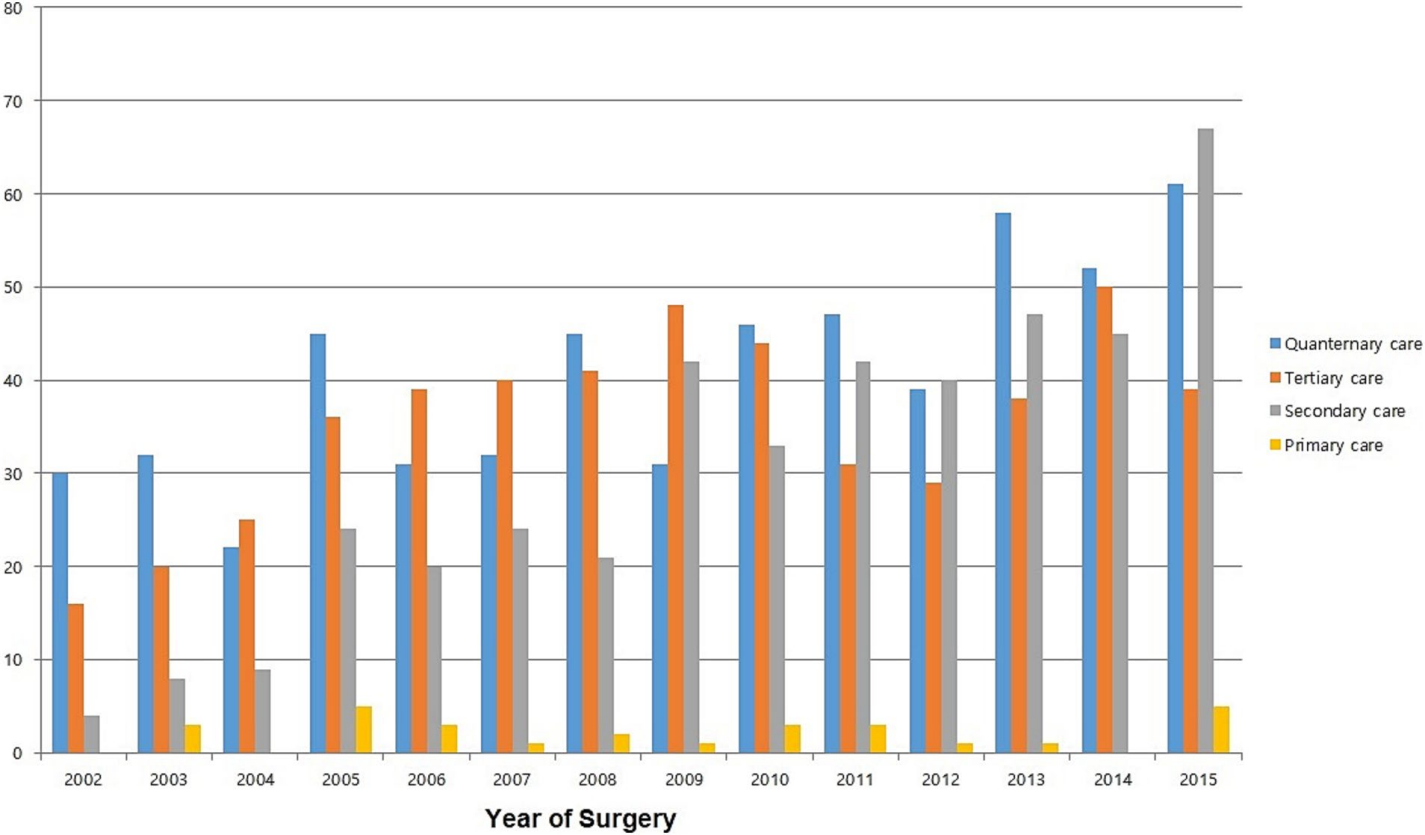
Trends in total hip arthroplasty according to the hospital type.

There was a notable shift in the types of hospitals performing THA. Between 2002 and 2008, most surgeries were performed in quaternary and tertiary care facilities. However, over time, the number of surgeries conducted in secondary care facilities increased (Figure 3).

Most of the baseline patient characteristics, including sex, height, weight, and BMI, were comparable between the two anesthesia groups (Table 1). However, an age difference was observed between the GA and RA groups; the GA group was younger. To mitigate intergroup differences, a 1:1 propensity score matching was conducted, resulting in a median value of 59 for both groups (*p* = 0.049).

**Table 1:** Characteristics of patients included in the study.

Characteristic	Pre-matching				Post-matching		
	Total (*N* = 1,522)	GA (*n* = 640)	RA (*n* = 882)	*p*-value	GA (*n* = 640)	RA (*n* = 640)	*p*-value
Age (years)	60.5 (50–70)	59 (47–69)	61 (51–71)	0.0054	59 (47–69)	59 (49–69)	0.049
Sex (male/female)	755/767	321/319	434/448	0.71	321/319	313/327	0.62
Elixhauser Comorbidity Score	4 (2–7)	4 (2–7)	4 (2–7)	0.82	4 (2–7)	4 (2–7)	0.98

Clinical characteristic	Pre-matching				Post-matching		
	Total (*N* = 1,212)	GA (*n* = 497)	RA (*n* = 715)	*p*-value	GA (*n* = 497)	RA (*n* = 513)	*p*-value
Height (cm)	160 (153–167)	160 (153–167)	160 (153–167)	0.85	160 (153–167)	160 (153–167)	0.59
Weight (kg)	61 (54–69)	61 (54–69)	61 (55–69)	0.77	61 (54–69)	61 (55–68)	0.83
BMI (kg/m^2^)	24.0 (21.85–26.2)	24.1 (21.6–26.4)	24.0 (21.9–26.1)	0.88	24.1 (21.6–26.4)	24.0 (22.1–26.0)	0.90

GA, general anesthesia; RA, regional anesthesia; BMI, body mass index. Data are presented as absolute numbers or medians (25th–75th percentiles). ^*^p < 0.05 for between-group comparisons.

When comparing the surgical outcomes between the GA and RA groups, the GA group had a younger average age and higher hospital costs. No significant differences were observed between the groups in terms of mortality, delirium, transfusion, or LOS. These findings remained consistent even after matching. Notably, the LOS was longer in the GA group, although no significant difference was observed (45 vs. 44 days in the RA group; *p* = 0.054; Table 2).

**Table 2:** Comparison of postoperative outcomes between the GA and RA groups before and after propensity score matching.

	Pre-matching				Post-matching			
	Total (*N* = 1,522)	GA (*n* = 640)	RA (*n* = 882)	*p*-value	GA (*n* = 640)	RA (*n* = 640)	*p*-value	95% CI
Mortality	11 (0.72)	6 (0.94)	5 (0.57)	0.54	6 (0.94)	4 (0.63)	0.75	0.14–2.81
Delirium	3 (0.20)	2 (0.31)	1 (0.11)	0.58	2 (0.31)	1 (0.16)	1.00	0.0085–9.60
Transfusion	1,233 (81.01)	519 (81.09)	714 (80.95)	0.94	519 (81.09)	515 (80.47)	0.78	0.74–1.26
LOS (days)	45 (33–62)	45 (33–67)	45 (32–61)	0.24	45 (33–67)	44 (32–59)	0.054	0.00–6.50
Hospital cost (won)	6,992,420 (6,237,660–8,713,860)	7,332,515 (6,328,980–9,668,620)	6,833,295 (6,170,910–8,139,150)	<0.0001	7,332,515 (6,328,980–9,668,620)	6,748,745 (6,115,870–8,089,100)	<0.0001	407,830–924,065

CI, confidence interval; GA, general anesthesia; RA, regional anesthesia; LOS, length of stay. Data are presented as absolute numbers or medians (25th–75th percentiles). ^*^p < 0.05 between-group comparisons.

The comparison of surgical complications between the anesthesia groups revealed that both before and after propensity score matching, the GA group showed a significantly higher incidence of ICU admission (61 vs. 27, *p* = 0.0002) and prosthesis failure (10 vs. 2, *p* = 0.039) than the RA group (Table 3). No significant differences in other complications were observed between the groups (Table 3).

**Table 3:** Comparison of postoperative complications between the GA and RA groups before and after propensity score matching.

	Pre-matching				Post-matching			
	Total (*N* = 1,522)	GA (*n* = 640)	RA (*n* = 882)	*p*-value	GA (*n* = 640)	RA (*n* = 640)	*p*-value	95% CI
ICU admission	109 (7.16)	61 (9.53)	48 (5.44)	0.0023	61 (9.53)	27 (4.22)	0.0002	0.26–0.68
Cardiac arrest	2 (0.13)	1 (0.16)	1 (0.11)	1.00	1 (0.16)	1 (0.16)	1.00	0.013–78.49
Myocardial infarction	13 (0.85)	4 (0.63)	9 (1.02)	0.41	4 (0.63)	5 (0.78)	1.00	0.27–6.30
Pneumonia	28 (1.84)	10 (1.56)	18 (2.04)	0.49	10 (1.56)	13 (2.03)	0.66	0.52–3.58
Stroke	31 (2.04)	14 (2.19)	17 (1.93)	0.72	14 (2.19)	15 (2.34)	0.85	0.52–2.22
Surgical site infection	35 (2.30)	14 (2.19)	21 (2.38)	0.80	14 (2.19)	12 (1.88)	0.69	0.39–1.85
Sepsis	10 (0.66)	6 (0.94)	4 (0.45)	0.34	6 (0.94)	3 (0.47)	0.51	0.081–2.34
Acute renal failure	6 (0.39)	2 (0.31)	4 (0.45)	1.00	2 (0.31)	2 (0.31)	1.00	0.072–13.79
Nerve injury	1 (0.07)	1 (0.16)	0 (0.00)	0.42	1 (0.16)	0 (0.00)		
Urinary tract infection	57 (3.75)	24 (3.75)	33 (3.74)	0.99	24 (3.75)	24 (3.75)	1.00	0.56–1.78
Prosthesis failure	14 (0.92)	10 (1.56)	4 (0.45)	0.025	10 (1.56)	2 (0.31)	0.039	0.021–0.94
Unplanned intubation	6 (0.39)	4 (0.63)	2 (0.23)	0.25	4 (0.63)	1 (0.16)	0.37	0.0051–2.53
Wound dehiscence	5 (0.33)	2 (0.31)	3 (0.34)	1.00	2 (0.31)	3 (0.47)	1.00	0.17–17.96
Other thromboembolic events	40 (2.63)	12 (1.88)	28 (3.17)	0.12	12 (1.88)	19 (2.97)	0.21	0.77–3.26
Intracranial hemorrhage	5 (0.33)	3 (0.47)	2 (0.23)	0.66	3 (0.47)	0 (0.00)		
Ventilator care	2 (0.13)	1 (0.16)	1 (0.11)	1.00	1 (0.16)	0 (0.00)		
Pulmonary thromboembolism	3 (0.20)	2 (0.31)	1 (0.11)	0.58	2 (0.31)	0 (0.00)		
Acute respiratory distress syndrome	5 (0.33)	3 (0.47)	2 (0.23)	0.66	3 (0.47)	1 (0.16)	0.62	0.0063–4.15
Pulmonary edema	4 (0.26)	0 (0.00)	4 (0.45)	0.14	0 (0.00)	3 (0.47)		
Hepatic failure	5 (0.33)	2 (0.31)	3 (0.34)	1.00	2 (0.31)	3 (0.47)	1.00	0.17–17.96

CI, confidence interval; GA, general anesthesia; RA, regional anesthesia; ICU, intensive care unit. Data are presented as absolute numbers, absolute numbers (percentages), or medians (25th–75th percentiles). ^*^p < 0.05 between-group comparisons.

## Discussion

In this study, we compared the effects of GA and RA on clinical outcomes in patients who underwent THA and found no significant differences in mortality or postoperative delirium between the two anesthesia methods. However, the ICU admission and prosthesis failure rates were significantly higher in the GA group. Other complication rates were not significantly different between the GA and RA groups. Notably, compared with RA, GA did not significantly prolong LOS but incurred higher costs.

From 2002 to 2015, the number of THA procedures steadily increased. Before 2008, the proportion of GA was comparable to that of RA. However, since 2009, RA has been performed more frequently than GA, indicating an increasing trend. A meta-analysis performed in 2006 by Mauermann et al. (11) indicated that patients undergoing THA under neuraxial anesthesia experienced reduced surgical time and decreased blood loss compared to those undergoing surgery under GA. In addition, the incidence of deep vein thrombosis and pulmonary embolism was significantly lower (11). Another meta-analysis reported that GA leads to a higher incidence of postoperative delirium than RA (12).

For these reasons, anesthesiologists’ preference for regional anesthesia in hip surgery has gradually increased (13, 14). However, despite the benefits of regional anesthesia, anesthesiologists may opt for general anesthesia if the patient refuses or poorly cooperates with regional anesthesia, expects long surgery durations, has coagulation disorders, or has structural cardiac dysfunction, among other factors (15). Since anesthesia selection based on these patient factors could lead to bias in this study, propensity score matching was performed based on the Elixhauser Comorbidity Score.

Over the years, a shift in the types of hospitals where surgeries are performed has been observed. Until 2005, the highest proportion of THA surgeries were performed in quaternary hospitals. However, from 2006, the highest proportion of THA surgeries were performed in tertiary care hospitals. From 2011, the number of THA surgeries performed in secondary and quaternary care hospitals were comparable. This phenomenon can be explained by the changes in national healthcare insurance policies and hospital management systems in South Korea.

In this study, no significant differences were observed in mortality, delirium, transfusion rate, and LOS between the two anesthesia groups. However, the ICU admission rate was more than twice as high in the GA group (9.53%) than in the RA group (4.22%). In addition, the incidence of prosthesis failure was higher in the GA group than in the RA group (1.56% vs. 0.31%, respectively). Nevertheless, these results should be interpreted with caution, owing to the potential of selection bias; because GA may have been administered more frequently in patients for whom surgery was expected to be challenging, eliminating this bias was difficult.

Previous studies have identified aseptic loosening and instability as the major causes of prosthesis failure (16, 17). A possible hypothesis for this occurrence is that while RA provides complete muscle relaxation during surgery (18), GA may offer a lower degree of muscle relaxation. Differences in the degree of muscle relaxation may contribute to variations in the loosening and instability rates after surgery (19). Additionally, a study comparing deep and moderate neuromuscular blockade in GA found that deep neuromuscular blockade reduced blood loss and was associated with lower levels of inflammatory markers and complication rates (19). However, another study suggested that a deep neuromuscular blockade in GA offers minimal benefit and increases the risk of complications (20). As RA provides superior muscle relaxation compared to GA, it may offer advantages in this regard.

This study was based on data obtained for individuals from South Korean; therefore, it may not be transferrable to Western and Asian populations. Specifically, based on Western epidemiological studies, total hip replacement occurs 3–25 times more frequently in Caucasians than in Asians (21). The difference may be attributable to genetic differences (21). In Western countries, over 75% of primary THA were performed for osteoarthritis, whereas the most common indication for primary THA in Asian countries was osteonecrosis of the femoral head (22). Osteonecrosis of the femoral head mainly affects young male adults in their third to fifth decade of life; this may explain why the rate of primary THA in Korea is consistently higher in men than in women, in contrast to that observed in Western countries. This also clarifies why 66% of patients treated with primary THA are under the age of 65 years, whereas most patients in Western countries are over 65 years old.

The average LOS for hip replacement in Korea is far longer than that in the United States (45 vs. 4.3 days) (23). Nevertheless, the average inpatient hospital charges for each procedure in Korea are approximately six times lower than those in the United States (22). South Korea has a universal healthcare system controlled by the government and managed by the National Health Insurance Corporation. Because medical care costs are tightly controlled by the government, a high supply of health and hospital services can be provided at low prices.

### Limitations

This study had some limitations. First, although propensity score matching was performed to adjust for age, sex, and comorbidities, age was still significantly different even after matching (*p* = 0.049). However, since both the GA and RA groups had a mean age of 59 years, this difference was statistically significant but clinically insignificant. Despite the use of advanced statistical techniques such as propensity score matching, the retrospective nature of the study carries inherent limitations. Second, because this study relied on claims data obtained from the NHIS-NSC database rather than clinical data, potential bias cannot be ruled out. Especially, data on the severity of pre-existing conditions were not available, which limits the ability to account for the potential influence of comorbidities on the outcomes. Third, since the study was conducted on Koreans, the results are specific to Koreans and the Korean healthcare system. Finally, although the sample size of this study was based on national health insurance data, it is still limited to a sample cohort. Expanding the sample size in future research would enhance the generalizability of the findings and allow for a more robust analysis of different patient subgroups. Therefore, caution is needed when interpreting the study results.

## Conclusion

Our study found that the different anesthesia types in patients who underwent THA resulted in comparable mortality and complication rates but required differing costs. This study relied on claim data obtained from the NHIS-NSC database rather than clinical data, potential bias cannot be ruled out. However, the strength of this study lies in the analysis of large and representative data rather than data from single institution.

## Data Availability

The datasets presented in this article are not readily available because this study was conducted within the limited network provided by the National Health Insurance Service. Therefore, data extraction outside of the study results is strictly prohibited, and access to the data is restricted. Requests to access the datasets should be directed to https://nhiss.nhis.or.kr/.

## References

[1] Tsertsvadze A Grove A Freeman K Court R Johnson S Connock M. Total hip replacement for the treatment of end stage arthritis of the hip: a systematic review and meta-analysis. PLoS One. (2014) 9:e99804. doi: 10.1371/journal.pone.0099804, PMID: 25003202 PMC4086719

[2] Fang M Noiseux N Linson E Cram P. The effect of advancing age on total joint replacement outcomes. Geriatr Orthop Surg Rehabil. (2015) 6:173–9. doi: 10.1177/2151458515583515, PMID: 26328232 PMC4536505

[3] Pivec R Johnson AJ Mears SC Mont MA. Hip arthroplasty. Lancet. (2012) 380:1768–77. doi: 10.1016/S0140-6736(12)60607-2, PMID: 23021846

[4] Neuman MD Rosenbaum PR Ludwig JM Zubizarreta JR Silber JH. Anesthesia technique, mortality, and length of stay after hip fracture surgery. JAMA. (2014) 311:2508–17. doi: 10.1001/jama.2014.6499, PMID: 25058085 PMC4344128

[5] Ahn EJ Kim HJ Kim KW Choi HR Kang H Bang SR. Comparison of general anaesthesia and regional anaesthesia in terms of mortality and complications in elderly patients with hip fracture: a nationwide population-based study. BMJ Open. (2019) 9:e029245. doi: 10.1136/bmjopen-2019-029245, PMID: 31501111 PMC6738684

[6] Bjørgul K Novicoff WM Andersen ST Brevig K Thu F Wiig M. No differences in outcomes between cemented and uncemented acetabular components after 12–14 years: results from a randomized controlled trial comparing Duraloc with Charnley cups. J Orthop Traumatol. (2010) 11:37–45. doi: 10.1007/s10195-010-0082-2, PMID: 20198405 PMC2837808

[7] Howie DW Holubowycz OT Callary SA. The wear rate of highly cross-linked polyethylene in total hip replacement is not increased by large articulations: a randomized controlled trial. J Bone Joint Surg Am. (2016) 98:1786–93. doi: 10.2106/jbjs.15.01248, PMID: 27807110

[8] Lee J Lee JS Park SH Shin SA Kim K. Cohort Profile: The National Health Insurance Service-National Sample Cohort (NHIS-NSC), South Korea. Int J Epidemiol. (2017) 46:e15. doi: 10.1093/ije/dyv319, PMID: 26822938

[9] Menendez ME Neuhaus V van Dijk CN Ring D. The Elixhauser comorbidity method outperforms the Charlson index in predicting inpatient death after orthopaedic surgery. Clin Orthop Relat Res. (2014) 472:2878–86. doi: 10.1007/s11999-014-3686-7, PMID: 24867450 PMC4117875

[10] Ondeck NT Bohl DD Bovonratwet P McLynn RP Cui JJ Grauer JN. Discriminative ability of Elixhauser’s comorbidity measure is superior to other comorbidity scores for inpatient adverse outcomes after total hip arthroplasty. J Arthroplast. (2018) 33:250–7. doi: 10.1016/j.arth.2017.08.032, PMID: 28927567

[11] Mauermann WJ Shilling AM Zuo Z. A comparison of neuraxial block versus general anesthesia for elective total hip replacement: a meta-analysis. Anesth Analg. (2006) 103:1018–25. doi: 10.1213/01.ane.0000237267.75543.59, PMID: 17000823

[12] Zhu X Yang M Mu J Wang Z Zhang L Wang H. The effect of general anesthesia vs. regional anesthesia on postoperative delirium—a systematic review and meta-analysis. Systematic review. Front Med. (2022) 9:844371. doi: 10.3389/fmed.2022.844371, PMID: 35419373 PMC8995788

[13] Memtsoudis SG Cozowicz C Bekeris J Bekere D Liu J Soffin EM. Anaesthetic care of patients undergoing primary hip and knee arthroplasty: consensus recommendations from the International Consensus on Anaesthesia-Related Outcomes after Surgery group (ICAROS) based on a systematic review and meta-analysis. Br J Anaesth. (2019) 123:269–87. doi: 10.1016/j.bja.2019.05.042, PMID: 31351590 PMC7678169

[14] Owen AR Amundson AW Fruth KM Duncan CM Smith HM Johnson RL. Spinal versus general anesthesia in contemporary revision total hip arthroplasties. J Arthroplast. (2023) 38:S184–S188.e1. doi: 10.1016/j.arth.2023.03.013, PMID: 36931357 PMC10334301

[15] Torrillo T. Chapter 121. Neuraxial anesthesia. In: Atchabahian A Gupta R, editors. The anesthesia guide. New York, NY: McGraw-Hill (2013)

[16] Dobzyniak M Fehring TK Odum S. Early failure in total hip arthroplasty. Clin Orthop Relat Res. (2006) 447:76–8. doi: 10.1097/01.blo.0000203484.90711.52, PMID: 16505710

[17] Melvin JS Karthikeyan T Cope R Fehring TK. Early failures in total hip arthroplasty—a changing paradigm. J Arthroplast. (2014) 29:1285–8. doi: 10.1016/j.arth.2013.12.024 24444568

[18] Scarborough RA. Spinal anesthesia from the surgeon’s standpoint. JAMA J Am Med Assoc. (1958) 168:1324–6. doi: 10.1001/jama.1958.03000100026005, PMID: 13587212

[19] Oh CS Lim HY Jeon HJ Kim TH Park HJ Piao L. Effect of deep neuromuscular blockade on serum cytokines and postoperative delirium in elderly patients undergoing total hip replacement: a prospective single-blind randomised controlled trial. Eur J Anaesthesiol. (2021) 38:S58–66. doi: 10.1097/EJA.0000000000001414, PMID: 33399376

[20] Curry C Steen K Craig W Cary CW Richard J Babikian G. Does deep neuromuscular blockade improve operating conditions during minimally invasive anterolateral total hip replacements?: A randomized controlled trial. Cureus. (2020) 12:e10328. doi: 10.7759/cureus.10328, PMID: 33052289 PMC7546586

[21] Oishi CS Hoaglund FT Gordon L Ross PD. Total hip replacement rates are higher among Caucasians than Asians in Hawaii. Clin Orthop Relat Res. (1998) 353:166–74. doi: 10.1097/00003086-199808000-00019, PMID: 9728171

[22] Yoon PW Lee YK Ahn J Jang EJ Kim Y Kwak HS. Epidemiology of hip replacements in Korea from 2007 to 2011. J Korean Med Sci. (2014) 29:852–8. doi: 10.3346/jkms.2014.29.6.852, PMID: 24932089 PMC4055821

[23] Kurtz SM Lau E Schmier J Ong KL Zhao K Parvizi J. Infection burden for hip and knee arthroplasty in the United States. J Arthroplast. (2008) 23:984–91. doi: 10.1016/j.arth.2007.10.017, PMID: 18534466

